# Hints on Vulcanite Work

**Published:** 1869-10

**Authors:** James B. Hodgkin

**Affiliations:** Alexandria, Va.


### AMERICAN JOURNAL

OF

### DENTAL SCIENCE.

Vol. III. THIRD SERIES—OCTOBER 1869. No. 6.

### ARTICLE I.

### *Hints on Vulcanite Work.*

By JAMES B. HODGKIN, D.D.S., Alexandria, Va.

The attention of the writer was called last winter to the improvements of Messrs. Stuck & Owens in vulcanite work, and being favorably impressed with its merits, a trial of it led to some experiments in varying the manipulations of the polished tin sheets used in that method, and also the block tin models, the results of which experiments are thought of sufficient importance to merit publication, as possessing advantages over the method of the above named gentlemen as set forth by them.

The advantages of the block-tin model are claimed to be "shrinkage," whereby a closer fit is obtained than can be by the ordinary plaster model. But the close observer will find so many vulcanite plates made on plaster models which give perfect satisfaction, as to doubt if the advantage of shrinkage is so great as is claimed in the main, however desirable it may be in certain exceptional cases. Allowing, however, that it is desirable to obtain a shrunken model, (as it unquestionably is occasionally) it is doubtful if the plates made after the directions of the patentees are as close-fitting as is claimed. Let any one carefully measurethe shrinkage of a tin model, and it will be found that it is about compen sated by the sheet of "chemically pure tin" which is burnished down over that model. It is doubtful if a plate made over a tin model thus prepared is any smaller than one made over a model of fine plaster.

There is, it may be said in passing, little or no advantage in making vulcanite plates as thin as those exhibited by the patentees; this, however, each operator can control for himself, and they are certainly no better for being highly polished on the plated surface, but the contrary.

The method adopted by the writer, after a careful trial of the method as devised by the gentlemen holding the above mentioned patent, is as follows: Take an impression in sand and plaster mixed in about equal proportions, using any means preferred to hasten the setting, a solution of half an ounce of Sulphate of Potash in a quart of water will answer for this purpose. An ordinary impression cup is used, which should be well polished. When the impression is fully set, saturate it with water, and it will, with a little manipulation, part from the cup. Tapping gently on the handle of the cup will often disengage it. This impression must be well dried, and the advantages of taking it thus are that there is no danger of melting cups, and consequently it relieves the operator from the anxious watching of the drying process, and obviates the necessity of sawing openings in the centre of the impression for the escape of steam to prevent "blowing." The method of Messrs. S. & O. involved the building across the back of the impression with plaster to keep in the melted metal. This is a waste of time, as ordinary putty answers a better purpose. In experimenting in this matter, the writer in following out the plan laid down by these gentlemen, melted several cups from inability to judge the temperature and in drying out the the model after their plan, which is done in the cup. The plan of taking the impression (the ordinary plaster one with the saw cuts in it, as per directions of Messrs S. & O.) out of the cup before drying it was tried, but it was found that building new plaster across the back part of the impression caused it to warp appreciably by its expansion in setting, and the plates made on these models rocked so that the impression of sand and plaster with putty surrounding it was found to be, in my hands at least, the best.

The model thus obtained is the one on which to vulcanize. No covering of sheet tin is wanted, and a decided shrinkage is obtained, but not too much when close adaptation is desired. The brilliant polish characteristic of the method under discussion, and which is so taking to the eye of the novice, is not obtained in so high a degree it is true, but for mouths where shrinkage is really needed a plate made directly on this model as above described, will be found to possess decided advantages over its more lustrous competitor. The model will come from the impression, if the latter is thoroughly dried, smooth and undefaced, and without the unseemly burs made by the saw-cuts in the impression, which are with difficulty effaced, and are usually covered by a vacuum cavity of huge dimensions.

Within the past few months I have placed in the mouths of patients a number of plates made after the manner above described, which are being worn with great and increasing satisfaction.

Many of these persons had spent much time and patience in vain endeavors to wear plates made after almost every other plan, both of metal and rubber; and all agree in pronouncing the present dentures much superior to anything heretofore worn. I may add that for several of them I had previously constructed rubber plates on several different methods, "Stuck & Owens" included, without success.

This much for a process, which I am of opinion, does not impinge the rights of the patentees mentioned above, and which I modestly think superior to theirs, and I may add if not an infringement, will not be patented, at least by myself.

I now proceed to notice what is considered to be an important modification of this process of using tin linings to the plaster models for forming rubber plates, a process quite as valuable as anything connected with the subject. To get a polished lingual surface to the plate where the ordinary plaster model is used,—in other words to be rid of nearly all the labor of finishing up rubber plates in either partial or full cases, the following method is adopted:

Take the impression in the usual method, and make the ordinary model. Form over this a loose plate of sheet gutta percha; sheet wax will not answer as well. Make this plate as thin as the vulcanite plate is desired to be. Mount up the teeth in the usual way. Insert the cast and teeth in the lower half of the flask, bringing the plaster over the cusps &c., as is usually practiced. When this has set, slightly oil the lingual surface of the base plate, and fill over with plaster,—in other words take a plaster impression of the exposed surface of the base-plate. When this has set, remove it and burnish down on it a piece of the pure sheet tin; trim this so that it will not reach quite to the teeth, it should, however, come back to the posterior edge of the plate. Place this on the base-plate and adjust it nicely into position, using the burnisher if necessary. It will not quite reach the teeth, and this is to allow the surplus rubber to pass out. When it is properly adjusted, put on the upper half of the flask and fill as usual, taking the usual precautions to ensure separation; cut vents for the escape of surplus rubber, as described below. Pack with rubber as usual, but before doing so polish this tin plate gently and thoroughly, being careful not to leave any soil on it, and especially taking pains that all traces of wax are removed, and being careful to keep the rubber scrupulously clean. If all has been well done and the flask entirely closed, it will be found after vulcanizing not more than one-eighth of an inch around the edge of the plate will require any finishing, and the labor of hours will have been reduced to almost minutes. Soft, half worn cotton cloth is best to polish this tin plate.

One other modification of this process, and this already too long article will be concluded. It applies to entire upper sets of plain teeth, with rubber above the teeth. The intelligent reader will perceive that the description just given applies to those cases where the teeth rest on the natural gums, or where there is rubber above the teeth: Let the teeth be mounted after any method preferred as to the sort of model used, either plaster or metal will answer. When the case is waxed up ready for investment, set the plate on the model and run a plaster cast over the lingual suface of the plate, getting also an accurate impression of the teeth. Put also a temporary investment about the outside of the teeth and plate above them, when this last has set pass a thin instrument through it at the median line, so that it may be taken off in two pieces without breaking it; when all is set firmly, remove the cast from the lingual surface, but do not disturb the outer investment. Burnish down on this impression the tin polishing plates as previously described, and fit it neatly and accurately to the plate and teeth, using a burnisher for this purpose; the outer investment will prevent the teeth being displaced. Now remove the temporary investment from the outside, it will come off in two pieces, separating where the division was made at the median line, and to each piece adapt a strip of the tin with fingers and burnisher; let them be slightly longer at the front than the cast on which it is moulded that they may lap a little. Adjust these strips neatly and closely to the necks of the teeth and to the base plate above them, seeing that they do not come much above where the rubber is to be. Insert this in the usual way, using the precautions to ensure cleanliness mentioned above, being careful to close the flask slowly and entirely. The product will be, in intelligent hands, a plate almost completely finished, a little trimming perhaps where the edges of the tin plate touch the teeth, and the removal of the waste rubber and finishing up the edge being all that remains to be done.

With regard to the vents for the escape of the surplus rubber, it may be mentioned that they are better if made by the removal of a small part of the entire surface of the plaster, instead of cutting grooves as is commonly done; cutting away the entire surface with a knife; commencing shallow near the teeth and deepening as the edge of the flask is approached, gives a freer vent to the surplus material and is equally good in other respects. But do not make the mistake of making this vent too wide next the teeth; in it rubber will pass out before it is slowly pressed into the desired place.

If what is written above shall on trial, prove as satisfactory to my fellows in practice as to myself, I shall consider myself recompensed for the writing of it in the "dog days" with a multitude of other cares upon me, and no time for revision.

---

