# Errata

**Published:** 1869-11

**Authors:** 


*Errata.*—In Dr. Hodgkin's article "Hints on Vulcanite Work," owing to a brief absence from home preventing a revise of proofs, we have the following errors to correct:

On page 252, ninth line from top, "plated" read "palatine." On page 254, seventh line from top, for "loose plate" read "base plate." On page 255, third line from top, after the words "there is," the word "no" has been omitted, the phrase being intended to read: "where there is *no* rubber above the teeth."

